# *TERT* Promoter C228T and C250T Hotspot Mutations Are Absent in *BRAF* V600E‐Positive Langerhans Cell Histiocytosis

**DOI:** 10.1002/cam4.71115

**Published:** 2025-07-29

**Authors:** Abdou Malik Da Silva, Zofia Hélias‐Rodzewicz, Irena Ungureanu, Jean‐François Emile

**Affiliations:** ^1^ Paris‐Saclay University, Versailles SQY University, EA4340‐BECCOH, Assistance Publique–Hôpitaux de Paris (AP‐HP), Ambroise‐Paré Hospital, Smart Imaging, Service de Pathologie Boulogne France

**Keywords:** *BRAF*, C228T mutation, C250T mutation, Langerhans cell histiocytosis, *TERT*

## Abstract

**Background:**

In various malignancies, the co‐occurrence of *BRAF* V600E and *TERT* promoter mutations (C228T and C250T) has been associated with tumor aggressiveness and poor prognosis. However, the presence of these *TERT* promoter mutations in Langerhans cell histiocytosis (LCH) remains unexplored.

**Methods:**

We investigated the prevalence of *TERT* promoter C228T and C250T mutations in 40 formalin‐fixed, paraffin‐embedded (FFPE) LCH samples positive for *BRAF* V600E. A nested PCR approach followed by Sanger sequencing, complemented by NGS, was used for mutation screening.

**Results:**

The variant allele frequency (VAF) of *BRAF* V600E in the analyzed samples ranged from 0.1% to 33.6%, with a median of 17%, and 33 of 40 successfully amplified LCH samples did not harbor *TERT* C228T or C250T mutations.

**Conclusion:**

Our findings indicate that LCH *BRAF* V600E‐positive samples lack the *TERT* promoter C228T and C250T hotspot mutations. This suggests that *TERT* promoter mutations may not play a significant role in LCH pathogenesis.

## 1 Introduction

Langerhans cell histiocytosis (LCH) is a myeloproliferative disorder caused by the proliferation in tissues or organs of histiocytes with structural and phenotypic characteristics of Langerhans cells, leading to a wide range of clinical manifestations [1, 2]. *BRAF* V600E is the most recurrent mutation identified in LCH, with prevalence ranging from 40% to 70% in patients [3, 4]. Co‐occurrence of *BRAF* and *TERT* mutations is a marker of poor diagnosis and compromises patient survival [5, 6]. Indeed, telomerase, by maintaining the telomere length at the extremity of chromosomes, promotes cellular immortality and tumorigenesis [7]. Two mutations in the proximal promoter of *TERT* termed as C228T and C250T (located at chr5: 1,295,228C>T and chr5: 1,295,250C>T respectively) are the most prevalently described [8, 9, 10]. It seems that these *TERT* promoter mutations generate novel binding sites in the gene promoter, thus could increase transcriptional activity up to fourfold [8]. *TERT* promoter mutations were detected with *BRAF* V600E in a wide range of human cancers, notably melanoma and thyroid cancer [9, 11, 12]. Although the oncogenic combination of *BRAF* V600E and *TERT* promoter mutations is considered a potent driver of human tumor aggressiveness [11], it was not previously explored in histiocytosis. Here, we aim to determine the occurrence of *TERT* promoter mutations, C228T and C250T, in LCH samples *BRAF* V660E. For this purpose, a nested PCR approach followed by Sanger sequencing previously described was used [10]. First, we assessed the efficiency of this approach for the detection of *TERT* promoter mutations by determining its sensitivity and testing its concordance in melanoma samples compared to a custom Taqman qPCR assay. In addition, next‐generation sequencing (NGS) was performed on a subset of samples, including those with low *BRAF* V600E variant allele frequency (VAF), to ensure sensitive detection of *TERT* promoter mutations beyond the limit of nested PCR–Sanger sequencing method.

## 2 Methods

The study was performed on formalin‐fixed, paraffin‐embedded (FFPE) blocks selected from LCH samples (*n* = 40) of patients from the biobank of the pathology department of Ambroise Paré Hospital. These samples showed at least 20% of tumor cellularity (Table 1), confirmed by pathologists and classified as LCH according to the WHO classification [1]. DNA was extracted from histiocyte‐rich areas using the Maxwell RSC DNA FFPE kit (Promega, Madison, WI, USA) following the manufacturer's recommendations. DNA extracts positive for the *BRAF* V600E mutation were identified by digital polymerase chain reaction with the QIAcuity Digital PCR system (Qiagen, Hilden, Germany). VAF was determined as the ratio of *BRAF* V600E to total *BRAF* alleles. These samples were screened for the C228T and C250T *TERT* mutations by using a nested PCR approach with primers and conditions previously described [9, 10]. Briefly, in a first PCR of 40 cycles, a 474‐bp product containing both C228T and C250T hotspot mutations in the proximal promoter region of *TERT* was amplified. For this, up to 23 ng of DNA was used. For non‐amplified samples, a second PCR of 40 cycles was performed on the first PCR product for amplification of a 163‐bp product. PCR products were sequenced on a Prism Model 3700 Capillary Array Sequencer (Applied Biosystems). Sequences obtained were compared with all sequences from the human reference genome (hg19) in the UCSC genome database (https://genome.ucsc.edu/), using BLAT [13]. For our efficiency study, three melanoma samples tested by the nested PCR approach, one positive for the C228T *TERT* promoter mutation, one positive for the C250T *TERT* promoter mutation, and one wild type were included. To evaluate the sensitivity, the C228T positive samples and the C250T positive samples were serially diluted into the wild‐type sample to yield mutant concentrations ranging from 6.25% to 50%. These diluted samples were tested twice by the nested PCR approach followed by Sanger sequencing. To evaluate the concordance, we used a custom Taqman Assay for C228T (Thermo Fisher Scientific; ANXHAWX), as the manufacturer failed for C250T (Thermo Fisher Scientific; ANWDGCZ). The C228T Taqman assay included a VIC fluorescence wild‐type probe, a FAM fluorescence mutation‐specific probe, and primer pairs to amplify the target sequence. Real‐time PCR was performed in a final reaction volume of 25 μL and consisted of 23 ng of DNA sample, 1× Taqman Genotyping Master Mix, and 1× Custom *TERT* mutation assay mix. Amplifications were run on a 7500 Real‐Time PCR system (Applied Biosystems, Foster City, CA, USA) at the following cycling condition: one cycle of 95°C for 10 min, then 50 cycles of 95°C for 15 s, and 62°C for 90 s. Allelic discrimination and amplification plots were performed using the 75000 software v2.3.

**TABLE 1:** Clinicopathological characteristics of LCH cases included in this study by anatomical location.

Location	Number of cases	Age (years)		Tumor cellularity (%)		VAF (%)	
		Median	Range	Median	Range	Median	Range
Bone	19	20.0	1.0–68.0	50	20–80	17.3	4.4–33.2
Skin	12	2.4	0.1–77.0	30	20–70	13.2	0.1–33.6
Digestive	2	54.0	47.0–61.0	55	50–60	15.4	12.0–18.8
Lymph node	2	3.0	1.0–5.0	45	20–70	14.1	3.2–25.0
Mucosa	2	20.5	2.0–39.0	40	40–40	18.9	11.8–26.0
Pleuropulmonary	2	62.5	61.0–64.0	55	50–60	13.4	6.7–20.0
Orbital	1	2.0	—	50	—	17.0	—

Finally, selected samples, including those with a *BRAF*V600E VAF below the detection limit of the nested PCR–Sanger sequencing approach, were analyzed by next‐generation targeted sequencing of the *TERT* promoter hotspot regions (C228T and C250T) using the Illumina MiSeq platform. Briefly, 52 histiocytosis samples with varying DNA quality were processed. DNA was qualified as described by the manufacturer and libraries were prepared using the Qiagen Targeted DNA Pro Procedure with a Custom Design Panel. Libraries were created with a unique molecular index to increase the sensitivity of this method. Sequencing data were analyzed using Qiagen CLC Genomics Software. Median coverage was 100 (ranging from 10 to 1158) and 64.5 (ranging from 16 to 731) at C228T (c.‐124C>T chr5:1295113) and C250T (c.‐146C>T chr5:1295135), respectively.

## 3 Results and Discussion

**FIGURE 1:**
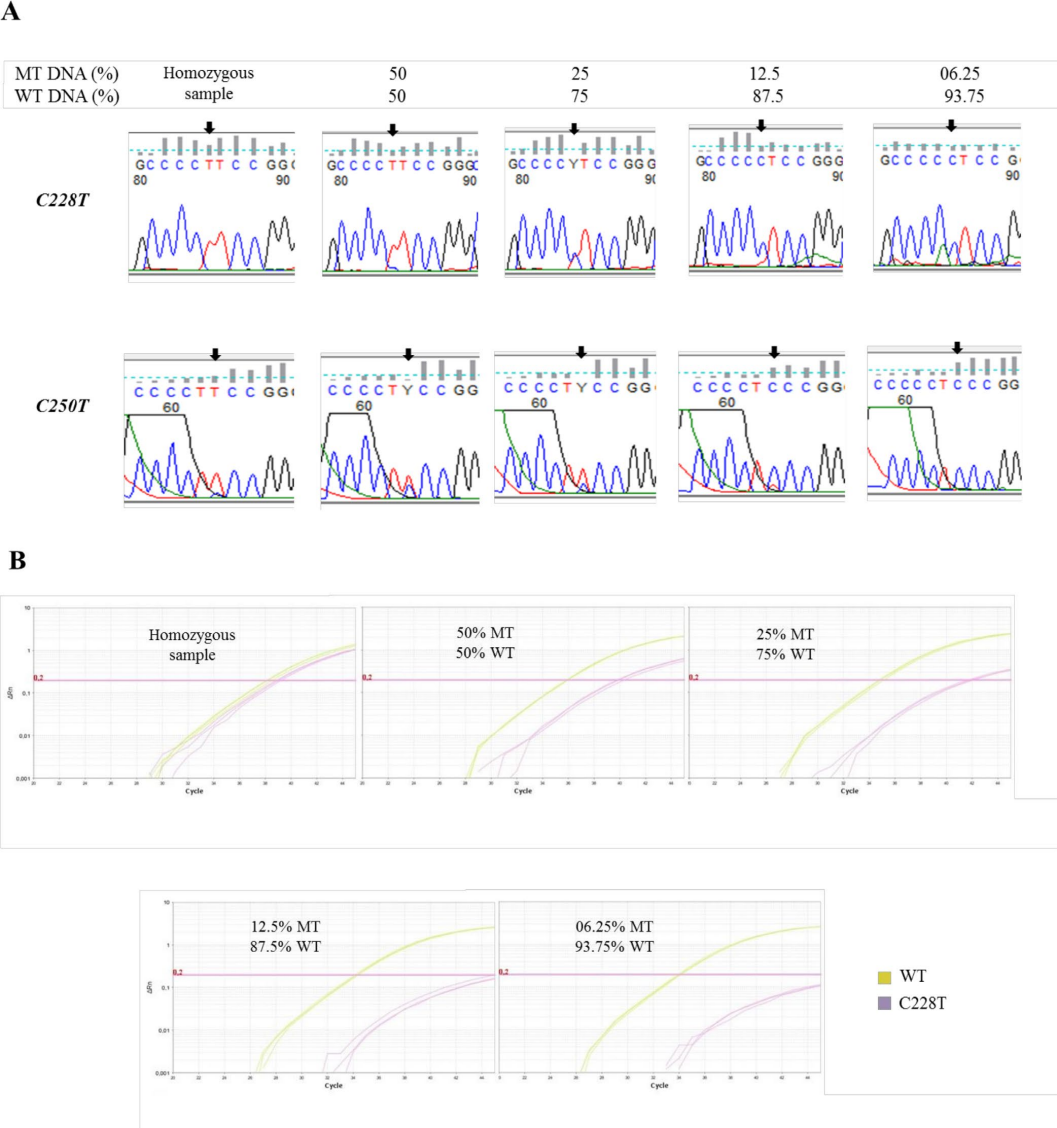
Sensitivity and concordance of the nested PCR–Sanger sequencing method used in this study with TaqMan assay for detection of *TERT* promoter mutations in melanoma samples. (A) Representative Sanger sequencing chromatograms from serial dilutions of melanoma DNA harboring C228T and C250T *TERT* promoter mutations into wild‐type DNA. Mutant allele frequencies tested include 50%, 25%, 12.5%, and 6.25%. Black arrows indicate the positions of C228T and C250T mutations on the chromatograms. The minimal detection threshold was determined to be 12.5% VAF. (B) Real‐time PCR amplification plots using a custom TaqMan assay for the C228T mutation. Samples with 25% and 50% mutant allele frequency show a distinct FAM fluorescence signal. No amplification was observed at 12.5% and below, confirming lower sensitivity compared to nested PCR. MT, mutant; WT, wild type.

For detection of the *TERT* promoter mutations, most of the previous studies used the Sanger sequencing approach, requiring a minimum mutant DNA frequency of 20% for positive screening [9, 14, 15, 16]. Here, the threshold limit of the nested PCR approach followed by Sanger sequencing was 12.5% for both C228T and C250T *TERT* variants contained in pooled melanoma DNA samples (Figure 1A). When the DNA mixtures contain a high frequency of the C228T allele (≥ 25%), both methods used were concordant and generated a positive signal (Figure 1A,B). However, the nested PCR approach showed higher detection sensitivity than the custom Taqman assay, as at a 12.5% allele rate, the C228T *TERT* mutation was detected by the nested PCR approach, while it failed by the Taqman assay (Figure 1A,B). VAF and amplicon size are probably the most important factors explaining differences in PCR‐based sensitivity [14]. It seems that the nested PCR substantially increases the VAF compared to the Taqman assay, thus improving performance detection for Sanger sequencing.

Previous studies reported a link between telomerase expression and activation of the MAPK pathway in melanoma, suggesting that *TERT* promoter mutations occurred significantly with *BRAF*/*NRAS* mutations [17, 18, 19]. This justifies our focus on investigating *TERT* promoter mutations in LCH with *BRAF* V600E positive. LCH *BRAF* V600E‐positive samples of 40 patients mostly originated from bone (*n* = 19) and skin (*n* = 12) (Table 1). The age of patients ranged from 0.1 to 77 years, with a mean of 25.3 years and the male/female ratio was 1.35:1. Of the 40 samples tested, seven failed the *TERT* promoter amplification. None of the 33 samples amplified and sequenced harbored *TERT* promoter C228T and C250T hotspot mutations. The *BRAF* V600E VAF ranged from 0.1% to 33.6% with a median value of 17% (Table 1). The VAFs of 10/33 (30.3%) amplified samples, ranging from 0.9% to 12%, were lower than our detection limit. These samples with low VAF were likely to be undetectable by our approach. In addition, the distribution of VAF in histiocytosis samples was previously described to be widely varied (ranged from 0.04% to 44% [20]). Therefore, we assume that the nested PCR approach followed by Sanger sequencing used in this study may underestimate the *TERT* promoter mutation prevalence. To address this limitation, 26 samples (all 10 with VAF below 12.5%, and 16 samples randomly selected) underwent targeted NGS analysis of the *TERT* promoter. None of these samples harbored C228T or C250T mutations, further supporting their absence in *BRAF* V600E‐positive LCH.

*TERT* promoter mutations were not frequently observed in some tumors [21, 22]. Furthermore, similarly to our data, in tumors such as lung cancer, gastric cancer, human B‐cell non‐Hodgkin lymphoma, and colorectal cancer, no *TERT* promoter mutations were detected, irrespective of *BRAF* mutation [22, 23, 24]. Therefore, the coexistence of *TERT* mutations and *BRAF* V600E is not prevalent. Upregulation of *TERT* expression can be due to mechanisms other than mutations in the gene promoter. *TERT* overexpression was demonstrated to be mediated by cooperation between *BRAF* V600E and transcription factors or by *TERT* promoter methylation [11, 25]. Telomerase activity in histiocytosis *BRAF* V600E positive could be investigated in view of these latter molecular events.

In conclusion, our results indicate that LCH *BRAF* V600E‐positive samples screened were devoid of C228T and C250T, the two most important *TERT* promoter mutations previously reported. This suggests that *TERT* mutations may be irrelevant to the pathogenesis of LCH.

## Author Contributions

**Abdou Malik Da Silva:** investigation, writing – original draft, methodology, data curation, writing – review and editing. **Zofia Hélias‐Rodzewicz:** conceptualization, investigation, writing – original draft, methodology, writing – review and editing, data curation, supervision. **Irena Ungureanu:** writing – review and editing, data curation, visualization. **Jean‐François Emile:** conceptualization, investigation, funding acquisition, methodology, supervision, resources, data curation, writing – review and editing.

## Ethics Statement

This study was approved by the Ethics Committee, Sud‐Ouest et Outre‐Mer II (#2019‐A01814‐53). All procedures implemented in studies involving human participants complied with the ethical standards of the institutional and/or national research committee and the 1964 Declaration of Helsinki and its subsequent amendments or comparable ethical standards. Written informed consent was obtained from all participants whose samples were included in the study.

## Conflicts of Interest

The authors declare no conflicts of interest.

## Data Availability

The data that support the findings of this study are available on request from the corresponding author. The data are not publicly available due to privacy or ethical restrictions.
